# Detection of breast cancer by ATR-FTIR spectroscopy using artificial neural networks

**DOI:** 10.1371/journal.pone.0262489

**Published:** 2022-01-26

**Authors:** Rock Christian Tomas, Anthony Jay Sayat, Andrea Nicole Atienza, Jannah Lianne Danganan, Ma. Rollene Ramos, Allan Fellizar, Kin Israel Notarte, Lara Mae Angeles, Ruth Bangaoil, Abegail Santillan, Pia Marie Albano

**Affiliations:** 1 Department of Electrical Engineering, University of the Philippines Los Baños, Los Baños, Laguna, Philippines; 2 Department of Biological Sciences, College of Science, University of Santo Tomas, Manila, Philippines; 3 Research Center for the Natural and Applied Sciences, University of Santo Tomas, Manila, Philippines; 4 The Graduate School, University of Santo Tomas, Manila, Philippines; 5 Mariano Marcos Memorial Hospital and Medical Center, Batac, Ilocos Norte, Philippines; 6 Faculty of Medicine and Surgery, University of Santo Tomas, Manila, Philippines; 7 University of Santo Tomas Hospital, Manila, Philippines; University of Houston, UNITED STATES

## Abstract

In this study, three (3) neural networks (NN) were designed to discriminate between malignant (*n* = 78) and benign (*n* = 88) breast tumors using their respective attenuated total reflection Fourier transform infrared (ATR-FTIR) spectral data. A proposed NN-based sensitivity analysis was performed to determine the most significant IR regions that distinguished benign from malignant samples. The result of the NN-based sensitivity analysis was compared to the obtained results from FTIR visual peak identification. In training each NN models, a 10-fold cross validation was performed and the performance metrics–area under the curve (AUC), accuracy, positive predictive value (PPV), specificity rate (SR), negative predictive value (NPV), and recall rate (RR)–were averaged for comparison. The NN models were compared to six (6) machine learning models–logistic regression (LR), Naïve Bayes (NB), decision trees (DT), random forest (RF), support vector machine (SVM) and linear discriminant analysis (LDA)–for benchmarking. The NN models were able to outperform the LR, NB, DT, RF, and LDA for all metrics; while only surpassing the SVM in accuracy, NPV and SR. The best performance metric among the NN models was 90.48% ± 10.30% for AUC, 96.06% ± 7.07% for ACC, 92.18 ± 11.88% for PPV, 94.19 ± 10.57% for NPV, 89.04% ± 16.75% for SR, and 94.34% ± 10.54% for RR. Results from the proposed sensitivity analysis were consistent with the visual peak identification. However, unlike the FTIR visual peak identification method, the NN-based method identified the IR region associated with C–OH C–OH group carbohydrates as significant. IR regions associated with amino acids and amide proteins were also determined as possible sources of variability. In conclusion, results show that ATR-FTIR via NN is a potential diagnostic tool. This study also suggests a possible more specific method in determining relevant regions within a sample’s spectrum using NN.

## Introduction

Breast cancer remains the most prevalent cancer among women. Biennial mammography has been highly recommended for women 50 to 74 years old for early detection of this disease. Sensitivity of mammography has been increased to 92.7% when combined with magnetic resonance imaging (MRI). Meanwhile, combination with ultrasound (US) can increase sensitivity to only 52%. Therefore, in high-risk women for whom supplemental screening is specified, MRI is recommended when possible [1]. Supplemental screening with US for women with intermediate risk and dense breasts is an option to increase cancer detection. The mammographic sensitivity for breast cancer in women with very dense breasts is 47.6% and increased to 76.1% with US screening [2]. Suspicious lesions detected during mammograms are usually biopsied to confirm or rule out breast cancer. The most common form of biopsy is the core needle biopsy (CNB), which involves the removal of a portion of a tumor for histologic evaluation. The remainder is removed later after a definitive diagnosis of cancer. However, since tissue samples are collected by three to nine passes with a biopsy needle, the incisional surgical procedures become associated with elevated incidence of lymph node metastasis and higher local recurrence rates [3]. CEA can also be analysed to screen for breast cancer. However, it lacks disease sensitivity and specificity, hence cannot be used for screening a subpopulation with high risk for malignancies, a general asymptomatic population, or for independently diagnosing cancer. CA 15–3, which are soluble forms of the transmembrane protein Mucin1 (MUC1), is said to be overexpressed in malignant breast tumors. It was suggested that CA 15–3 and CEA can be considered complementary in detecting recurrence of breast cancer. However, their sensitivity is low and independent of the majority of the prognostic parameters that may be considered before relapse [4].

The potential of using infrared spectroscopy, in particular Fourier transform infrared (FTIR) spectroscopy, has been gaining popularity for cancer diagnostics over the last few years. The distinctive spectral properties associated with the changes in chemical composition and structure of biomolecules can be recognized by FTIR spectroscopy, making it a potential diagnostic tool [5]. Hence, when cells or tissues undergo transformation from normal to cancerous, changes in the physico-chemical structures and properties in a variety of their biomolecules can be simultaneously and indiscriminately probed by FTIR spectroscopy [6, 7]. FTIR, as compared to traditional microscopic examination of hematoxylin and eosin (H&E) stained biopsies, is more rapid, cost-effective, and objective since reading is based on changes in biochemical properties instead of morphology [7]. It eliminates the possibility of intra- and inter-observer variability, which is often the problem with H&E staining. Moreover, this technology does not make use of dyes and other contrasting agents which may interfere or affect the reading. Hence, it provides more accurate and reproducible results.

The application of artificial intelligence (AI) in cancer diagnosis is no longer new; numerous studies have already been applied to address the most prevalent cancers such as lung cancer [8–10], thyroid cancer [11], ovarian cancer [12, 13] and breast cancer [14–17]. Most studies make use of image-based data such as MRI images, computed tomography (CT) scans, positron emission tomography (PET) scans, X-rays, and H&E-stained biopsy images, which is the gold standard [18]. The most successful AI implemented in these studies involves artificial neural networks (NN), in particular, convolutional neural networks (CNN) due to their proven effectiveness in processing images. Furthermore, the underlying architecture of a CNN makes it easily feasible to create visual representation, highlighting the site of malignancy within a medical image. What limits image-based AI diagnostics, however, is that they heavily rely on the detection of a visible abnormality within the scanned region. This implies that a patient may already be at an advanced stage of malignancy before possible detection. Moreover, the presence of dyes and other contrasting agents may make it difficult to apply in other laboratories an AI model trained using the procedures in one laboratory, if protocols and procedures are not well standardized.

The appeal of using FTIR data in AI is that they come in less file size and are easier to process than images, while still providing sufficiently adequate information for samples [19]. Hence, data storage costs may be minimized significantly and models utilizing such data become easier and faster to train, hence minimizing training costs. Moreover, the use of FTIR data may be able to predict the onset of cancer even before evident morphological changes [5], hence addressing the limitation of image-based AI diagnostics. However, since NNs are essentially black boxes, the underlying process involving its decision-making is inherently unknown; thus making them less appealing to use in a clinical setting. Furthermore, FTIR data are less intuitive to interpret than images, even with the assistance of AI visualizations; making them difficult to interpret. In this study, a method was formulated to address this limitation by providing a novel process of determining the most prevalent biomarkers as seen by trained artificial NNs; hence providing a basis on decision-making process. The proposed method is a modification of NN perturbation-based sensitivity analysis [20–22], which probes a NN’s sensitivity towards changes in an input variable.

Hence, this study showed the potential of artificial neural networks (ANN) in accurately diagnosing breast cancer through infrared spectroscopy. Specifically, it designed multiple ANN to diagnose malignancy from breast tissues using ATR-FTIR data. The classification performance of the NN models were compared to six (6) most widely-used machine learning models. Moreover, this study also proposed a novel method for determining the IR regions which may be significant in determining breast cancer malignancy, as seen by a NN design. This proposed method may serve as a baseline process in analysing spectral data, and more importantly provide new insights and directions for pathologists and medical practitioners.

## Materials and methods

### Ethical clearance

Ethical clearance was obtained from the Institutional Review Board (IRB) of the University of Santo Tomas Hospital (USTH) in Manila, Philippines (Ref. No.: IRB-2018-07-135-IS) and Mariano Marcos Memorial Hospital and Medical Center (MMMH-MC) in Ilocos Norte, Philippines (Ref. No.: MMMHMC-RERC-15-006). Written informed consent from the participants or their legal guardians have been waived by the respective ethics review boards since the study was restricted to the use of archived formalin fixed paraffin embedded (FFPE) breast tissues and did not involve additional procedures nor pose risk of harm to subjects. All methods were carried out in accordance with the Declaration of Helsinki and its later amendments.

### Study population and sample preparation

Two hundred (200) FFPE breast biopsies obtained from 192 adult patients seen at USTH and MMMH between January 2016 to December 2016 were included in the study. The samples were diagnosed by the resident pathologists of the hospital study sites as either benign (*n* = 91) or malignant (*n* = 101) based on microscopic examination of H&E-stained biopsies. The malignant samples were further subclassified as invasive ductal carcinoma, residual ductal carcinoma-in-situ, or invasive lobular carcinoma; and the benign samples as fibroadenoma, fibrocystic disease, benign fibroadipose tissue, fibrocollagenous cyst wall, or intraductal papillomas.

The FFPE tissues were uniformly cut at 5-μm thickness using a microtome (Leica Biosystems, Germany) and three (3) adjacent tissue sections were mounted on glass slides. The two (2) outer sections were stained with H&E for re-evaluation by a third-party pathologist who was blinded of the original diagnosis. The pathologist was instructed to classify the biopsy sample as either benign or malignant and to mark the location of the cancer cells if the sample was malignant, to serve as guide in the ATR-FTIR analysis [23]. The inner or middle tissue section was deparaffinized with xylol, dehydrated with alcohol, rinsed in distilled water, dried overnight, and subjected to ATR-FTIR analysis [24, 25].

Only the samples with similar diagnosis by the resident pathologists of the hospital study sites and the third-party pathologist were considered for further analysis. In this case, out of the 200 archival samples, only 166 (*n* = 78 benign; *n* = 88 malignant) were taken for ATR-FTIR processing. Furthermore, each of the 166 samples corresponded to one patient each to satisfy a one-is-to-one correspondence between patients and specimens [11].

### ATR-FTIR spectral analysis

An FTIR mid-infrared spectrometer equipped with a platinum ATR single reflection diamond sampling module (Bruker Optics, Germany) was used to obtain spectra of the breast samples. A performance qualification (PQ) test using OPUS 8.0 software’s fully automated validation program was initially performed to ensure quality and accuracy of spectral data. The deparaffinized breast tissue sections were positioned directly in contact with the ATR diamond crystal’s surface (2 mm x 2 mm) and the mid-IR region of 4000 cm^-1^ to 600 cm^-1^ was passed to and from the ATR accessory. Spectra were generated at a spatial resolution of 4 cm^-1^ and an average of 48 scans was co-added to obtain an adequate signal-to-noise ratio [26–28], which was further supported by the software’s validation program as “acceptable”. Prior to scanning each tissue sample, the background spectrum was recorded, and this spectrum was systematically subtracted by the software to routinely eliminate atmospheric effects. The malignant samples were scanned along the area containing the cancer cells, while the benign samples were scanned at random spots throughout their entire tissue section. The spectral data associated with a benign or a malignant tissue sample was obtained by computing for the median spectrum of their respective 48 scans.

### Characterization and pre-processing of spectral data

The spectral data set, *X*_*SD*_, consisted of *N* | *N* = 166 spectral vectors ${\vec{x}}_{SD}^{\left(i\right)}\in {\mathbb{R}}^{L}\;\forall \;i\in \mathbb{N}\leq N$, comprising of 78 malignant FTIR data, $X_{SD}^{\left(M\right)}\subset X_{SD}$, and 88 benign FTIR data, $X_{SD}^{\left(B\right)}\subset X_{SD}$, where ℒ | ℒ = 462 denotes the length of each vector. An element of a vector ${\vec{x}}_{SD}$, ${\vec{x}}_{SD}\left(j\right)\forall \;j\in \mathbb{N}\leq L$ corresponds to an absorbance reading within the fingerprint region of 1800 cm^-1^ to 850 cm^-1^ at 2 cm^-1^ steps. Furthermore, *X*_*SD*_ can also be characterized as a matrix of ℝ^*N*×*ℒ*^ dimension.

All obtained spectral data were internally normalized using z-score normalization, which is the recommended method of normalization for the FNN designs [29, 30]. The normalization was done to eliminate bias from *y*-value discrepancies among the IR samples. Here, normalization was done per ${\vec{x}}_{SD}^{\left(i\right)}$ using the equation

$${\vec{x}}_{SD}^{\left(i\right)}≔\frac{{\vec{x}}_{SD}^{\left(i\right)}\;-mean\left({\vec{x}}_{SD}^{\left(i\right)}\;\right)}{std\left({\vec{x}}_{SD}^{\left(i\right)}\;\right)}\;\forall \;i\in \mathbb{N}\leq N$$ (1)

where the *mean*() and the *std*() notations denote the mean and the standard deviation of the elements of the vector ${\vec{x}}_{SD}^{\left(i\right)}$. The implemented normalization scales the elements of ${\vec{x}}_{SD}^{\left(i\right)}$ to have an overall mean of 0, and a standard deviation of 1. *X*_*SD*_ also underwent baseline correction via OPUS 8.0 software via “rubber band method” with 64 baseline points. This was done to approximate a polynomial fit based on the minima of *y*-values of each element vector ${\vec{x}}_{SD}^{}\in X_{SD}$. The fitted polynomial was then deducted for all ${\vec{x}}_{SD}^{}$ to extract the baseline corrected spectrum [25, 31–34]. Finally, the corrected spectrum was scaled within the fingerprint region, from 1800 cm^-1^ to 850 cm^-1^ [32, 35]. Other than baseline correction using rubber band method, no further user intervention was done to assess the spectral data. To visualize the average spectrum of benign and malignant breast tissues, their respective median values for each wavenumber was plotted.

### Principal component analysis (PCA)

Principal component analysis (PCA) was performed to visualize the distribution of benign and malignant samples over two of the PCA’s most dominant components (*F*_1_ and *F*_2_). The process of translating *X*_*SD*_ to the reduced variable space, *X*_*PCA*_ (*X*_*SD*_ *→ X*_*PCA*_) is given by the equation

$$X_{PCA}=\left(X_{SD}-{\bar{X}}_{SD}\right)\times \left[{\vec{s}}_{F1}{}^{T},{\vec{s}}_{F2}{}^{T}\right]$$ (2)

where *X*_*PCA*_ ∈ ℝ^*N* × 2^ is the reduced sample space, ${\bar{X}}_{SD}$ is the mean absorbance value of ${\vec{x}}_{SD}^{\left(i\right)}\in X_{SD}\;\forall \;i\leq N$, and ${\vec{s}}_{F1}$ and ${\vec{s}}_{F2}$ are the eigenvectors corresponding to the largest eigenvalue of the covariance matrix $S_{SD}={\left(X_{SD}-{\bar{X}}_{SD}\right)}^{T}\times \left(X_{SD}-{\bar{X}}_{SD}\right)$. A PCA biplot of the malignant and the benign samples were drawn along the *F*_1_ and *F*_2_ axes to visualize the sample distribution.

### Classifiers

Three (3) feed forward neural networks (FNN) of different layer sizes were designed in the study. To benchmark the NN models, six of the most widely used machine learning models were also created, in particular, linear discriminant analysis (LDA), support vector machine (SVM), logistic regression (LR), decision tree (DT), random forest (RF), and Naïve Bayes (NB). The following subsections discuss in detail the design of each model.

#### Cross-validation of models

A 10-fold cross-validation procedure was used to evaluate all models; where 70% of the spectral data set *X*_*SD*_, were used for the training set *S*_*TR*_ ⊂ *X*_*SD*_, and the remaining 30% were equally partitioned for the validation set *S*_*V*_ ⊂ *X*_*SD*_ (15%) and the test set *S*_*TS*_ ⊂ *X*_*SD*_ (15%). The cross-validation procedure was repeated over 50 trials (*T*) to ensure stability of results [36]. For each trial, the elements of the sets *S*_*TR*_, *S*_*V*_, and *S*_*TS*_ were reselected from *X*_*SD*_ randomly. The sets satisfied the criteria $S_{TR}^{\left(i\right)}\;\cup S_{V}^{\left(i\right)}\;\cup \;S_{TS}^{\left(i\right)}=X_{SD}$ and $S_{TR}^{\left(i\right)}\;\cap \;S_{V}^{\left(i\right)}\cap \;S_{TS}^{\left(i\right)}=\;\emptyset \;\;\forall \;i\in \mathbb{N}\leq T$. Moreover, the ratio of malignant and benign samples was preserved for each set. To evaluate the performance of each model, the metrics area under the curve (AUC), accuracy (ACC), positive predictive value (PPV), negative predictive value (NPV), recall rate (RR) and specificity rate (SR) were obtained. The overall mean and standard deviation of the metrics over the 50 trials were obtained using the formulas

$$M_{m}=\;\frac{1}{T}\overset{T}{\underset{1}{\sum }}\left(\frac{1}{N}\overset{N}{\underset{1}{\sum }}m_{n,t}\right)$$ (3.1)

$$\Sigma_{m}=\frac{1}{T}\overset{T}{\underset{1}{\sum }}\left(\sqrt{\frac{\sum_{1}^{N}{\left(m_{n,t}-{\bar{m}}_{n,t}\right)}^{2}}{N-1}}\right)$$ (3.2)

in which *M*_*m*_ and *Σ*_*m*_ are the overall mean and the overall standard deviation of a metric *m*, where *m*_*n*,*t*_ is the metric value of a metric *m*, for a trial *t* ∈ ℕ ≤*T* and a fold *n* ∈ ℕ ≤ 10. Moreover, the variable ${\bar{m}}_{n,t}$ in Eq 3.2 is the *N*-fold mean of a metric *m*, which is also equal to $\frac{1}{N}\overset{N}{\underset{1}{\sum }}m_{n,t}$ from Eq 3.1.

All models were designed and implemented using MATLAB R2020b on an Intel i7-6700 3.40 GHz CPU and an Nvidia GeForce GTX 1050 Ti GPU over a 16 GB RAM.

#### Feedforward neural networks (FNN)

Three (3) feed forward neural networks (FNN) were designed in the study. Each neural network has an input layer ${\vec{N}}_{i}$, consisting of ℒ nodes corresponding to the length of each spectral vector input ${\vec{x}}_{SD}$, and an output layer ${\vec{N}}_{o}$, consisting of 2 nodes which correspond to the sample’s diagnosis of being benign or malignant. A neural network varies in hidden layer size (*n* = 2, *n* = 4, *n* = 8) with respect to the other. For all FNNs, scaled exponential linear units (SELU) were used as activation functions within the hidden layers [37], and softmax function for the output layer.

#### Linear discriminant analysis (LDA)

Here, *S*_*TR*_ was used to design an LDA model *f*_*LDA*_(*x*) which returns the probability of a spectral vector input $x:=\;{\vec{x}}_{SD}$ from being benign or malignant. *S*_*TS*_ was used to measure the metric performance of the model. Before constructing a linear separator among the samples, principal component analysis (PCA) was performed to reduce the dimension of data from 462 to two variables, which are *F*_1_ and *F*_2_. The reduction of dimensional space via PCA (*X*_*SD*_ → *X*_*PCA*_) follows the discussion from Eq 2.

The LDA model was constructed following Fisher’s criterion [38] where the probability density function describing the likelihood of a sample from being malignant or benign is given by the function *f*_*LDA*_(*x*) [11]

$$f_{LDA}\left(x\right)=softmax\left(\left[p_{M}\left(x\right),\;\;p_{B}\left(x\right)\right]\right)$$ (4.1)

in which *p*_*M*_(*x*) and *p*_*B*_(*x*) are normal probability density functions describing the probability distribution of malignant and the benign samples, respectively. The normal curves are defined as

$$p\left(x\right)=\;\frac{1}{\sigma \sqrt{2\pi }}e^{-\frac{{\left(\gamma \left(x\right)-\mu \right)}^{2}}{2\sigma^{2}}}$$ (4.2)

where $\gamma \in \Gamma \;|\;\Gamma =w_{min}^{T}\;X_{PCA}$, where *Γ* is the projection of *X*_*PCA*_ onto *w*_*min*_, while μ and *σ* are the class mean and standard deviation, respectively.

In evaluating an element of the test set ${\vec{x}}_{SD}^{\left(TS\right)}\in S_{TS}$, the ℒ × 2 matrix $\left[{\vec{s}}_{F1}{}^{T},{\vec{s}}_{F2}{}^{T}\right]$ obtained from the training set via Eq 2 was first used to translate ${\vec{x}}_{SD}^{\left(TS\right)}$ from a dimension of ℝ^ℒ^ to ℝ^2^ before evaluation using Eq 4.2.

#### Support vector machine (SVM)

The designed SVM is a linear SVM of input $x:={\vec{x}}_{SD}$. The SVM was designed from the elements of the training set by considering an unconstrained Langrange optimization problem given by the equations [39, 40]

$$f\left(\vec{w},b\right)=\frac{1}{2}{\left|\vec{w}\right|}^{2}$$ (5.1)

$$g\left(\vec{w},b\right)=y_{i}\left(x\cdot \vec{w}+b\right)-1$$ (5.2)

$$L_{\min\left(\vec{w},b\right)}\left(\vec{w},b\right)=f\left(\vec{w},b\right)-\overset{N_{TR}}{\underset{i=1}{\sum }}\alpha_{i}g\left(\vec{w},b\right)=\frac{1}{2}{\left|\vec{w}\right|}^{2}-\;\overset{N_{TR}}{\underset{i=1}{\sum }}\alpha_{i}\left[y_{i}\left(x\cdot \vec{w}+b\right)-1\right]$$ (5.3)

where $f\left(\vec{w},b\right)$ corresponds to the equation for maximizing support vector elements separation, $g\left(\vec{w},b\right)$ is the conditional function for clustering the malignant and the benign classes, and L_min(*w*,*b*)_ is the Langrangian *L* of $f\left(\vec{w},b\right)$ and $g\left(\vec{w},b\right)$ of variables $\vec{w}$ and *b*. Here, $\vec{w}$ is the weight vector of ℝ^ℒ^ dimension, *b* is the bias of ℝ^1^ dimension, while $\alpha_{i}\in \alpha |\;\alpha \in {\mathbb{R}}^{N_{TR}\times L}$ is the SVM’s *α*-matrix where the *α*_*i*_ ≠ 0 ∀ *i* ∈ ℕ ≤ *N*_*TR*_ elements correspond to the SVM’s support vectors. To determine suboptimal values for $\vec{w}$, *b*, and *α*, stochastic gradient descent (SGD) was implemented by considering the gradients

$$\frac{\frac{1}{2}{\left|\vec{w}\right|}^{2}}{\partial \vec{w}}=\frac{\frac{1}{2}\vec{w}\cdot \vec{w}}{\partial \vec{w}}$$ (5.4)

$$\frac{\partial L\left(\vec{w},b\right)}{\partial \vec{w}}=\vec{w}-\overset{N_{TR}}{\underset{i=1}{\sum }}\alpha_{i}y_{i}x$$ (5.5)

$$\frac{\partial L\left(\vec{w},b\right)}{\partial b}=-\overset{N_{TR}}{\underset{i=1}{\sum }}\alpha_{i}y_{i}$$ (5.6)

To optimize the model, a grid search was performed from a series of learning rates *ℓ* from 1 to 5×10^−5^ over 1000 epochs. The validation set accuracy was considered as the optimization metric which determined the superiority of one model from the other. The process was repeated for 50 trials to ensure stability. The average validation accuracy of a model over the 50 trials determined the overall metric of the model for a considered learning rate, *ℓ*_*i*_. The *ℓ*_*i*_ which constituted the highest overall validation accuracy was considered as optimal learning rate to train and test the SVM model. The output probability diagnosis of the model for benign and malignant cases, *p*_*SVM*_(*x*), was computed using Platt’s method [41].

#### Logistic regression (LR)

The designed LR model is a ℒ-input classifier with an output probability *p*_*LR*_(*x*) quantifying the likelihood of an input $x:={\vec{x}}_{SD}$ to be malignant. *p*_*LR*_(*x*) is defined as

$$p_{LR}\left(x\right)=\frac{e^{b+\vec{w}\cdot x}}{1+\;e^{b+\vec{w}\cdot x}}$$ (6.1)

where *w* ∈ ℝ^ℒ^ and *b* ∈ ℝ^1^ are the weights and bias characterizing the LR model. In training the model, SGD was used to minimize loss over the training set. The considered loss function was the binary cross-entropy loss function given by

$$H=-\;\frac{1}{N}\overset{N}{\underset{i=1}{\sum }}y_{i}\cdot \log\left(p\left(y_{i}\right)\right)+\left(1-y_{i}\right)\cdot \text{log}\;\left(1-p\left(y_{i}\right)\right)$$ (6.2)

where *p*(*y*_*i*_) denotes the probability of obtaining a malignant diagnosis given a theoretical output of *y*_*i*_; where *y*_*i*_ = 1 for malignant cases, and *y*_*i*_ = 0 for benign. To optimize the model, a grid search was performed with a design similar to that performed for the SVM.

#### Decision tree (DT) and random forest (RF)

The classification and regression trees (CART) algorithm was used to generate DTs of binary splits. The Gini’s diversity index was used to find the best input *Φ*_*j*_ | *j* ∈ ℕ ≤ ℒ for splitting the training set for each iteration of branching; where *Φ*_*j*_ is the *j*^*th*^ wavenumber from the fingerprint region 1800 cm^-1^ to 850 cm^-1^. Gini’s diversity index [42] is given by

$$GINI{\left(j\right)}_{t}=\overset{2}{\underset{m=1}{\sum }}\left(\frac{N_{m|\hat{\Phi }}}{N_{t}}\cdot \left(P{\left(m|t\right)}^{2}+{\left[1-P\left(m|t\right)\right]}^{2}\right)\right)$$ (7.1)

where the maximum bound for the summation operation denotes the binary characteristic of the splitting considered. Furthermore, $N_{m|\hat{\Phi }}$ is the total number of class *m* separated by the value $\hat{\Phi }\in {\vec{\Phi }}_{j}|{\vec{\Phi }}_{j}=row_{j}(X_{SD})$ at node *t*, *N*_*t*_ is the total number of elements in ${\vec{\Phi }}_{j}$ at node *t* and *P*(*m*|*t*) is the probability of class *m* for being either malignant or benign from happening at node *t*. Since the elements in ${\vec{\Phi }}_{j}$ are continuous variables, the best value of separation $\hat{\Phi }$ was identified from ${\vec{\Phi }}_{j}$ by considering the element having the least *GINI*(*j*)_*t*_ metric [43]. The branching was recursively performed for each newly created node until the performance in the validation set accuracy decreased.

The designed RF utilized the creation of trees following the previously discussed. The diagnosis of the RF was determined as the prevailing diagnosis made by its constituent bags of DTs. To determine the optimum number of trees *N*_*RF*_ for the RF, a grid search from 3 to 100 trees was performed. The validation set accuracy was considered as the optimization criteria of the search. Each simulation was repeated over 50 times for each iteration *n*|*n* ∈ {3 ≤ ℕ ≤ 100} to ensure stability. The average accuracy over the 50 trials served as the final performance metric of the RF for an *N*_*RF*_ equal to *n*. The final RF constituted the design with the highest average accuracy.

#### Naïve bayes (NB)

The designed NB is a classifier of two classes of *n*|*n* ∈ ℕ ≤ ℒ inputs. For each *j*^*th*^ input, ${\vec{\Phi }}_{j}$, the best $\hat{\Phi }$ value of separating the elements of ${\vec{\Phi }}_{j}$ between two sub-classes was determined. The algorithm for finding $\hat{\Phi }$ is the same as that of the DT and RF designs where the Gini’s index was used (Eq 7.1). The predictive value *f*_*NB*_(*x*) of the NB is defined as the probability of a sample for being malignant, given an input $x:={\vec{x}}_{SD}$. *p*_*NB*_(*x*) is given by

$$p_{NB}\left(x\right)=\frac{\prod_{j=1}^{n}P(m_{j}|malignant)}{\prod_{j=1}^{n}P\left(m_{j}\right)}$$ (8.1)

where the numerator corresponds to the total probability of an input *x* from happening, given *n*-inputs considered, with an *x*(*j*) value classified as class *m*_*j*_ for a determined separation ${\hat{\Phi }}_{j}$ for malignant cases. On the other hand, the denominator is the total probability of the set of *m*_*j*_ from *x* for ever happening. The NB classifier outputs a malignant diagnosis when *p*_*NB*_ > 50%; otherwise, the diagnosis is benign. In order to determine the optimal *n*-value for the classifier, the number of inputs was increased from 3 to ℒ, where the inputs of the least *GINI*(*j*)_*t*_ value were considered first. The optimization was terminated at the *n*-value where the validation accuracy of the model started to decrease. Each iteration of *n* was repeated for 50 trials, where the average validation accuracy from the 50 trials was the considered optimization metric criterion. The final NB constituted to the design with the highest average validation accuracy.

### Identification of dominant spectral components

In order to identify the most significant wavenumbers which influenced a sample’s diagnosis via the NN models, a novel sensitivity analysis was performed based on the optimized FNNs (*n* = 2, *n* = 4, *n* = 8). It must be noted that a visual peak analysis of the obtained spectral data was also performed prior to sensitivity analysis to compare the identified significant wavenumbers from the NN.

#### Visual peak analysis

Significant peaks in the fingerprint region were identified through visual inspection of *X*_*SD*_. Test of normal distribution using Shapiro-Wilk test and variance of homogeneity were performed for the identified peaks. Since all data followed a non-normal distribution, they were subjected to Mann-Whitney *U* test to assess if the absorbance peaks of malignant samples were significantly different (*p*-value <0.05) from the benign samples. Statistical analyses were performed using MATLAB 2020b.

#### Sensitivity analysis of neural network

A modified neural network committee-based (NNC) sensitivity analysis was considered using the input perturbation algorithm. In order to simplify the NNC sensitivity analysis, the committee of NNs were designed and trained following the design architecture of the optimized FNNs [20–22].

For the analysis, an experimental set *S*_*EXP*_ ⊂ *X*_*SD*_ was used to train and analyse the FNN design. *S*_*EXP*_ comprises of 70% randomly-selected elements from *X*_*SD*_. The elements of *S*_*EXP*_ was randomly selected from *X*_*SD*_. Moreover, the quantity of malignant and benign samples from *S*_*EXP*_ were equally proportioned. For each selected input ${\vec{x}}_{SD}\left(j\right)\forall \;j\in \mathbb{N}\leq L$, a perturbation Δ*x*_*j*_ from –50% to 50% of $\bar{{\vec{x}}_{SD}}\left(j\right)$ at 5% steps was added to the ${\vec{x}}_{SD}\left(j\right)$, and the mean square error (*MSE*) of the output was tabulated; where $\bar{{\vec{x}}_{SD}}\left(j\right)$ is the mean value of *S*_*EXP*_ for the *j*^*th*^ input [21]. The *MSE* for the *j*^*th*^ input variable at its *k*^*th*^ step perturbation Δ*x*_*j*,*k*_ (*MSE*_*j*,*k*_) is calculated using the formula

$$MSE_{j,k}=\overset{N_{EXP}}{\underset{l=1}{\sum }}\frac{\sum_{i=1}^{2}\sqrt{O_{j,k}{\left(i\right)}^{2}-\hat{O}{\left(i\right)}^{2}}}{2}$$ (9.1)

where *N*_*EXP*_ is the total number of elements in *S*_*EXP*_, *O*_*j*,*k*_ is the output vector of the model for Δ*x*_*j*,*k*_, and $\hat{O}$ is the ideal output vector; where $\hat{O}=\langle 1,0\rangle$ for a benign sample, and $\hat{O}=\langle 0,1\rangle$ for a malignant one. The overall response of the network *MSE*_*j*_ for the *j*^*th*^ input was computed by averaging *MSE*_*j*,*k*_ for all Δ*x*_*j*,*k*_ *k*-steps given by

$$MSE_{j}=\frac{\sum_{i=1}^{N_{k}}MSE_{j,k}\left(i\right)}{N_{k}}$$ (9.2)

To ensure stability of the performed sensitivity analysis, the process was repeated for a committee of 50 NN (*i*.*e*., 50 trials). The overall response of the *j*^*th*^ input, was computed as the average of the *j*^*th*^ input response over the 50 trials ${\bar{MSE}}_{j}$.

To visualize the perturbation response of the considered FNN, ${\bar{MSE}}_{j}$ was plotted for all *j* ∈ ℕ ≤ ℒ. The sensitivity analysis was performed for each FNN (*n* = 2, *n* = 4, *n* = 8).

#### Motivation and theory

The diagnostic ability of often-used machine learning models such as SVM, LDA, and PCA greatly relies on the variation among and between data within a data set *X*. This variation is often quantified using a covariance matrix, $S\in {\mathbb{R}}^{N^{2}}$. By obtaining the eigenvectors associated with *S*, the characteristic *form* of the data may be easily represented. However, this approach becomes ineffective if the variation among the data set elements *X*_*j*_ ∈ *X* becomes significantly small (but not infinitesimally small to imply repetitive data). In such a scenario: S → 0, the obtained eigenvector solutions, ${\vec{e}}_{j}|\;j\in \mathbb{N}\leq N$, become trivial where $\;\left|{\vec{e}}_{j}\right|\to 0\;\forall \;j\in \mathbb{N}\leq N$. This makes it difficult to distinguish important variables which may prove significant in determining an accurate diagnosis. For artificial NNs, the weights determine the correlation of the input parameters toward the outputs under 0-bias condition. Here, the magnitude of a weight determines the magnitude of the correlation, while the *sign* of the weight determines the direction of influence [44]. This simplistic model does not however explain the contribution of biases and activation functions in a network’s decision-making process.

The proposed model of this study probes the significance of an input parameter ${\vec{x}}_{SD}\left(j\right)$ based on the magnitude of change at the output for given ranges of input perturbations Δ*x*_*j*,*k*_. The magnitude of influence of an input variable is given by the *MSE*_*j*,*k*_, which is a magnitude value at the range of 0 to 1 since the output are probabilities. In obtaining *MSE*_*j*,*k*_, $X_{SD}^{\left(M\right)}$ and $X_{SD}^{\left(B\right)}$ were assumed as a single set since analyzing them separately would not provide an overall determination of the significant variables considering both classification. Furthermore, since the spectral data set *X*_*SD*_ was normalized for each set element ${\vec{x}}_{SD}^{\left(i\right)}$, *MSE*_*j*,*k*_ was expected to be less varied between samples for all ${\vec{x}}_{SD}^{\left(i\right)}\in X_{SD}$ across all input variables. This assumption makes it justifiable to denote the overall average, *MSE*_*j*_, as the overall measure of an input response for a single neural network. The overall *MSE* magnitude, ${\bar{MSE}}_{j}$, was assumed as the final metric to measure the influence of a given variable, which is the average *MSE*_*j*_ measure considering multiple similar NNs. In such process, it was assumed that each NN had similar input responses since each followed the same architecture, training, and optimization. Lastly, in determining the most significant input variables, ${\bar{MSE}}_{j}$ was no longer ranked in contrast to usual sensitivity analyses [20–22, 45, 46]. Since the functional groups and vibrational modes associated with the input variables are usually presented in ranges within the IR spectrum, it was more appropriate to identify and discuss significant peaks from the plot of ${\bar{MSE}}_{j}$ rather than in a ranked form.

**Fig 1:**
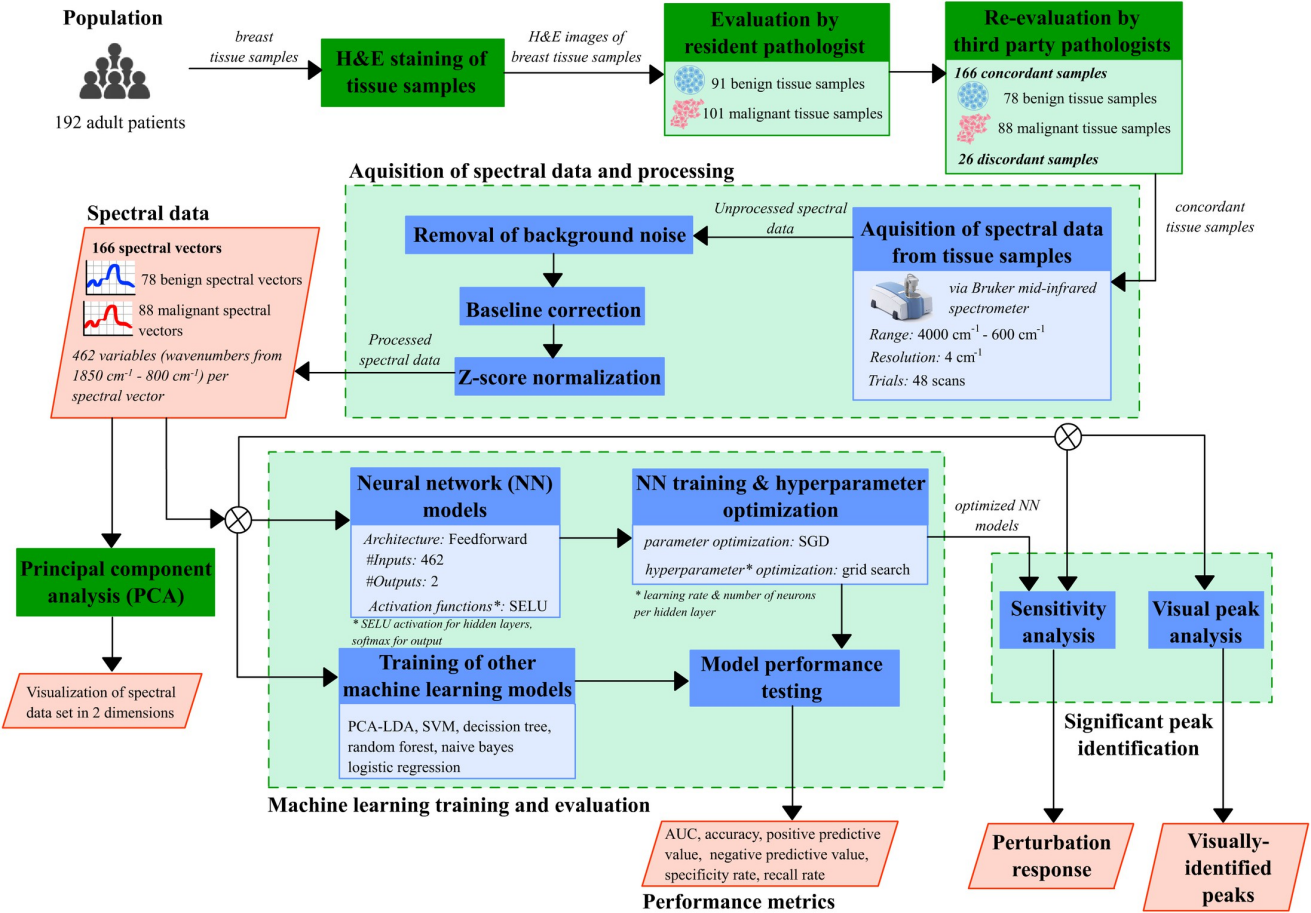
Experimental design process flowchart. The figure shows the experimental design implemented in the study, from the acquisition of breast tissue samples, to the acquisition, processing and analysis of spectral data.

Overall, this study proposed that input variables associated with comparatively high *MSE* constitute to significant wavenumbers important in the NN’s diagnosis. Since the NN models were assumed as the prevailing models and are highly accurate, the determined wavenumbers may thus provide insights in the associated changes in chemical composition and structure of biomolecules in cancerous breast tissues. The overall method implemented in the study is summarized in Fig 1.

## Results

### Samples

The clinical characteristics of the samples were retrieved from medical records and histopathology reports of the hospital study sites (Table 1). Among the malignant samples, majority were invasive ductal carcinoma. Meanwhile, the benign breast samples were mainly fibroadenoma and fibrocystic change (Table 1). The above classifications were based on microscopic examination of H&E-stained specimens and immunohistochemical staining (if needed or available) following the current WHO classification.

**Table 1:** Clinical data of the patients with breast lesions*.

Diagnosis	Classification*	Total no. of samples (*n* = 164)
Malignant	Invasive ductal carcinoma (*n* = 76)	88
	Others (*n* = 12)	
Benign	Fibroadenoma (*n* = 46)	78
	Fibrocystic change (*n* = 20)	
	Other (*n* = 12)	

*Retrieved from hospital records and were based on microscopic evaluation of H&E-stained slides by resident pathologists of the hospital study sites.

**Fig 2:**
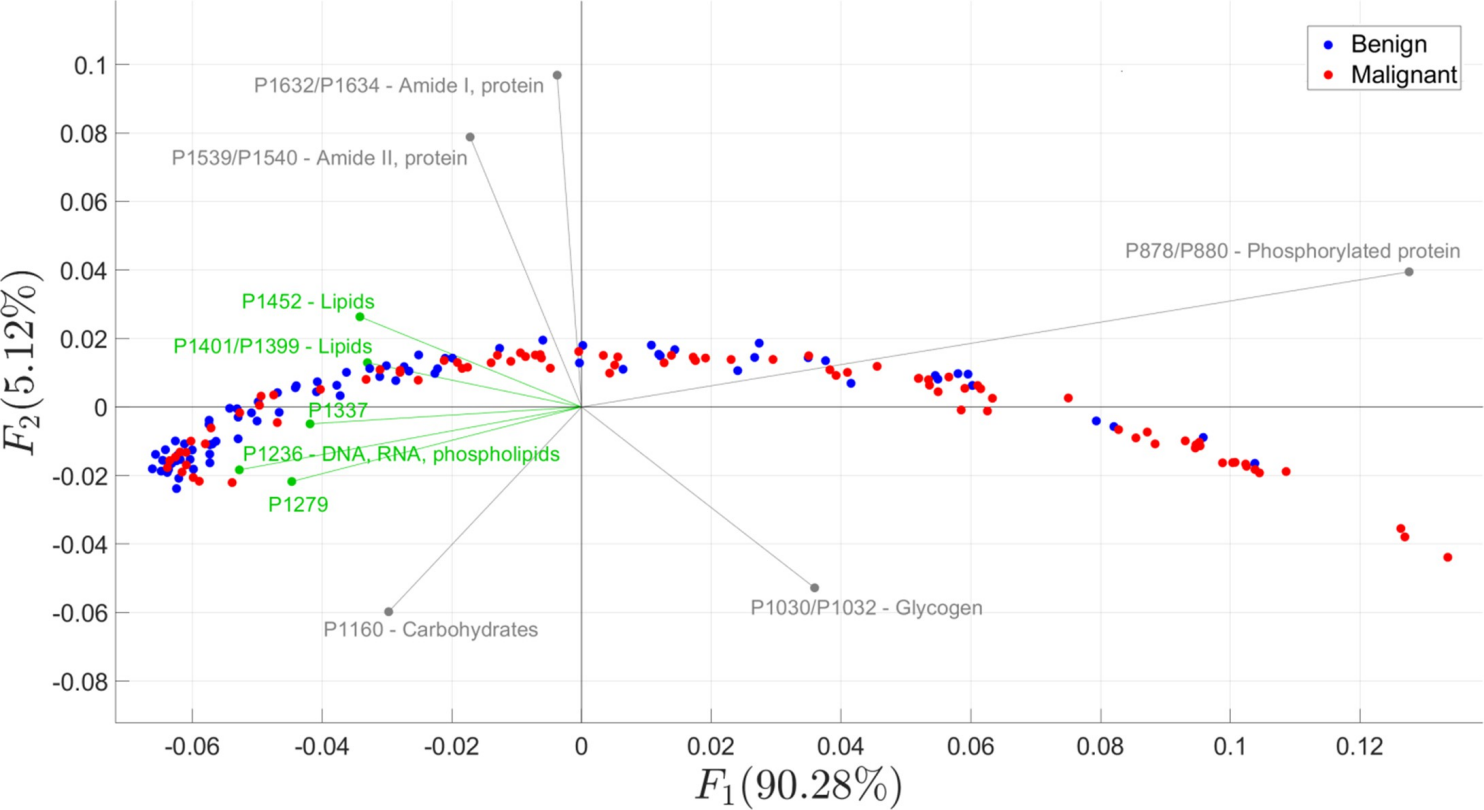
PCA biplot showing data points of malignant and benign samples. The red points denote malignant samples while blue points denote benign samples plotted across the two most dominant components (*F*_1_ = 90.28% and *F*_2_ = 5.21%). The vectors show the wavenumbers associated with peak absorbance, where those highlighted in green were identified as significant wavenumbers in discriminating benign from malignant samples.

The variation between benign and malignant samples is shown in Fig 2. From the PCA plot, 90.28% of the variability was associated with the first principal component *F*_1_, while only 5.12% was associated with the second principal component *F*_2_. Most of the benign samples were scattered along the negative domain of the *F*_1_ axis while malignant samples were evenly scattered. Both sample classes followed a parabolic distribution across the determined principal axes. Overall, the PCA biplot suggests that the benign and malignant breast samples were highly similar in characteristics.

### Feedforward neural network designs

The NN input layer consisted of 462 nodes which corresponded to the defined IR absorbance of each sample in frequencies between 1800 cm^-1^ to 850 cm^-1^. Three (3) FNN models were designed with varying layer sizes (*n* = 2, *n* = 4, *n* = 8). The quantity of neurons per FNN hidden layer was kept constant for each repetition. The general NN architectures are summarized in Table 2.

**Table 2:** Feedforward neural network architecture.

	FNN2	FNN4	FNN8
**Overall architecture**	input layer– 462 neurons	input layer– 462 neurons	input layer– 462 neurons
	2 hidden FC layers –350 neurons	4 hidden FC layers– 400 neurons	8 hidden FC layers –300 neurons
	output layer– 2 neurons	output layer– 2 neurons	output layer– 2 neurons
**Learning rate**	0.01	0.01	0.01
**Network weight initialization**	Gaussian random: *σ* = $\sqrt{\frac{1}{input\;size}}$, *μ* = 0		
**Input normalization**	Z-score normalization: $\vec{x}:=\frac{\vec{x}-mean\left(\vec{x}\right)}{std\left(\vec{x}\right)}$		
**Activation functions**	Input/Hidden layers–SELU		
	Output layer—softmax		
**Cost/fitness function**	Binary cross-entropy		
**Fully connected layer dropout**	90% SELU dropout		
**Training optimizer**	AdaGrad stochastic gradient descent (SGD)		
**Epoch**	1000		

Gaussian random initialization was assumed for weight initialization, while a zero-value initialization was used for the biases. Moreover, SELU activation was used for all neuron activation functions except for the respective output layers in which the softmax activation function was used. All NN were trained through backpropagation via AdaGrad stochastic gradient decent (SGD) [47] over 1000 epochs. The binary cross-entropy function was considered as the cost function for all NN designs during the training process. To avoid over-fitting, a dropout of 90% was used for each feed forward hidden layers as recommended for SELU activation [29, 37].

### Neural network optimization

Pre-training, a grid search was performed to determine the optimal learning rate and the layer width. To limit the search space of the performed grid search, the explored learning rates *L*, and the considered layer width *N*_*W*_, were limited to 10 and 20 elements, respectively, where

$$L\;∋\;l_{i}=\frac{l_{i-1}}{k}|l_{0}=1;\;i\in \mathbb{N}\leq 9\;;k=\{\begin{array}{c} 2\;\forall \;\;i\in {\mathbb{N}}_{Odd}\\ 5\;\forall \;\;i\in {\mathbb{N}}_{Even} \end{array}$$

and *N*_*W*_ = {10, 15, 20, 25, 30, 40, 50, 60, 80, 100, 120, 140, 160, 180, 200, 250, 300, 350, 400, 462}. For every combination of hyperparameters, each NN model was trained over 1000 epochs using the training set via a 10-fold cross-validation procedure over 50 trials. The best hyperparameter combination for each model was identified as the hyperparameter combination with the highest average validation accuracy.

**Fig 3:**
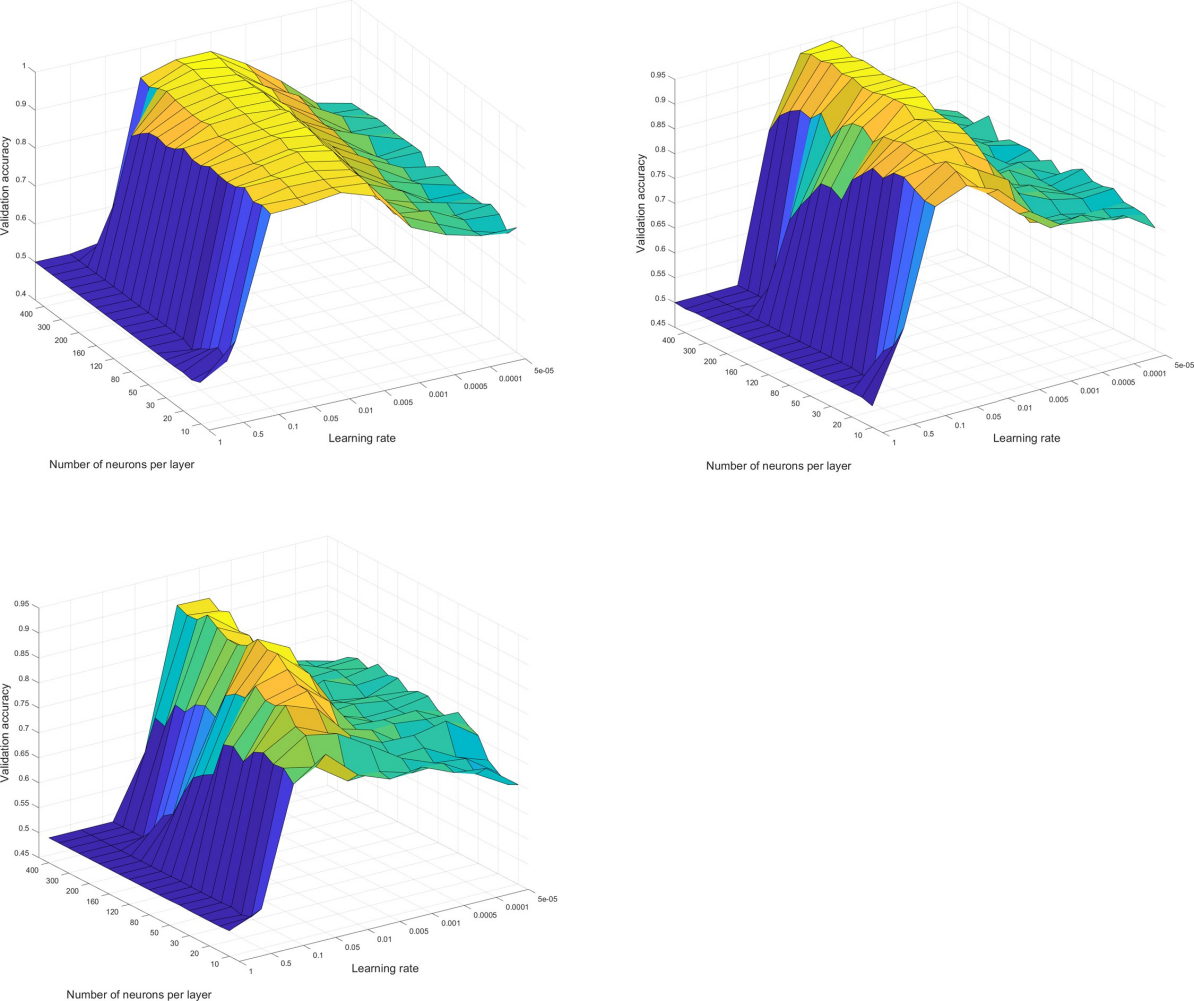
**A. Grid search surface plot of FNN2.** The plot shows the grid search surface plot for optimizing the FNN2 model for learning rate and number of neurons per hidden layer. Validation accuracy peaked at a learning rate of 0.01 and 350 neurons per hidden layer. Low performance at high learning rates (blue region) may be due to divergence and high parameter oscillations, while stagnation of performance at low learning rates (green region) may be due to insufficient training time. **B. Grid search surface plot of FNN4.** The plot shows the grid search surface plot for optimizing the FNN4 model for learning rate and number of neurons per hidden layer. Validation accuracy peaked at a learning rate of 0.01 and 400 neurons per hidden layer. The same behavior in the low and high learning rate regions, observed in the FNN2 surface plot, is also evident here. **C. Grid search surface plot of FNN8.** The plot shows the grid search surface plot for optimizing the FNN8 model for learning rate and number of neurons per hidden layer. Validation accuracy peaked at a learning rate of 0.01 and 300 neurons per hidden layer. Among the FNN surface plots, the FNN8 constituted to the most unstable response in validation accuracy.

The result of the grid search for each NN design (Fig 3) shows that for all models, the designs peaked at a learning rate of about 0.005 to 0.1, with the FNN2 having the most stable deviation of performance across its range and the FNN8 as the most unstable. Regardless of the model, it can further be seen that at learning rates above 0.1, the models exhibited very poor performance in validation accuracy which may be due to divergence and large weight oscillations. Meanwhile, the validation accuracy stagnated at learning rates of below 0.005, which may be due to very small changes in the NN’s weights and biases. The optimized learning rate for each model was identified to be all equal to 0.01, while the number of neurons per layer of each model to achieve best performance were 350, 400, and 300 for FNN2, FNN4, and FNN8, respectively.

### Diagnostic performance of models

Diagnostic performance of the NN models and the other machine learning models (NB, DT, RF, LR, LDA, SVM) was determined by comparison with the gold standard, which is the microscopic examination of H&E-stained tissues by pathologists. In terms of ACC, NPV and RR, all the NN models were able to significantly surpass all the other machine learning models (Tables 3 and 4). However, none of the models was able to outperform the SVM model as to AUC (95.72% ± 4.94%). Among the NN models, the best AUC was achieved by FNN4 (90.48% ± 10.30%) followed by FNN8 (90.35% ± 10.10%) then FNN2 (90.05% ± 9.95%). The relatively low AUC among NN models may be attributed to a lack of training time. In terms of PPV, the NN model performances did not significantly differ from the best benchmark model, which was SVM. As to NPV, the NN models were able to surpass the performance of the best benchmark model by an average of 4.42% for FNN2, 3.16% for FNN4 and 2.08% for FNN8. As to SR, the models performed significantly less than SVM, but were able to outperform the other benchmark models. The models were able to outperform SVM in terms of RR by an average of 2.94% for FNN2, 1.23% for FNN4 and 0.17% for FNN8. Note, however, that the RR value for FNN8 was not significantly differed from that of the SVM. The observed increase in metric performance from FNN8 to FNN2 may be due to a lack in training time for the deeper models. Overall, the significantly high ACC value of the FNN models may prove them viable classifiers in distinguishing malignant from benign samples using FTIR data. The results are summarized in Tables 3 and 4.

**Table 3:** Diagnostic performance of the models.

Model	AUC (%)	Accuracy (%)	PPV (%)	NPV (%)	SR (%)	RR (%)
**LDA**	71.58±11.84	66.17±10.20	77.67±13.47	55.98±15.34	74.35±11.80	61.56±9.65
**Logistic Regression**	86.27±9.58	65.05±12.63	68.83±31.57	60.79±41.06	53.46±32.44	73.00±23.96
**Naïve Bayes**	66.90±10.90	64.69±10.41	54.44±16.24	77.40±15.09	60.23±9.62	74.22±15.28
**Decision Trees**	69.03±12.83	69.38±11.58	69.56±15.82	69.56±17.37	67.79±13.58	73.19±12.93
**Random Forest**	84.63±9.30	76.78±9.49	76.94±14.56	76.60±14.88	76.18±12.23	79.91±10.88
**SVM**	95.72±4.94	90.44±7.78	91.03±9.61	89.77±10.33	90.56±9.68	91.40±8.34
**FNN2**	90.05±9.95	96.06±7.07	89.83±12.57	94.19±10.57	85.56±18.33	94.34±10.54
**FNN4**	90.48±10.30	95.54±8.07	91.72±12.06	92.93±12.81	88.38±17.47	92.63±13.31
**FNN8**	90.35±10.10	95.46±7.55	92.18±11.88	91.85±12.67	89.04±16.75	91.58±13.47

**Abbreviations:** *LDA*–linear discriminant analysis; *SVM*–support vector machines, *FNN*—feed forward neural network; *AUC*—area under the curve; *PPV*—positive predictive value, *NPV*- negative predictive value; *SR*—specificity rate; *RR*—recall rate.

**Table 4:** Test of significance of neural network performance metrics relative to SVM.

Model	Difference of average performance metric* (p-value**)					
	AUC (%)	ACC (%)	PPV (%)	NPV (%)	SR (%)	RR (%)
**FNN2**	-5.67 (*<< 0*.*05*)	5.62 (*<< 0*.*05*)	-1.20 (*0*.*2137*)	4.42 (*<< 0*.*05*)	-4.95 (*0*.*0219*)	2.94 (*0*.*0006*)
**FNN4**	-5.24 (*<< 0*.*05*)	5.09 (*<< 0*.*05*)	0.69 (*0*.*5249*)	3.16 (*0*.*0001*)	-2.18 (*0*.*0161*)	1.23 (*0*.*0359*)
**FNN8**	-5.37 (*<< 0*.*05*)	5.01 (*<< 0*.*05*)	1.15 (*0*.*1042*)	2.08 (*0*.*0036*)	-1.52 (*0*.*1814*)	0.17 (*0*.*5832*)

*The differences of performance metrics were computed by subtracting the FNN performance metric to that of the SVM.

** Mann-Whitney *U* test (two-tailed); significant when *p*<0.05.

**Abbreviations:** **:** *LDA*–linear discriminant analysis; *SVM*–support vector machines, *FNN*—feed forward neural network; *AUC*—area under the curve; *ACC—*accuracy *;PPV*—positive predictive value, *NPV*- negative predictive value; *SR*—specificity rate; *RR*—recall rate.

### Visual peak analysis of data

**Fig 4:**
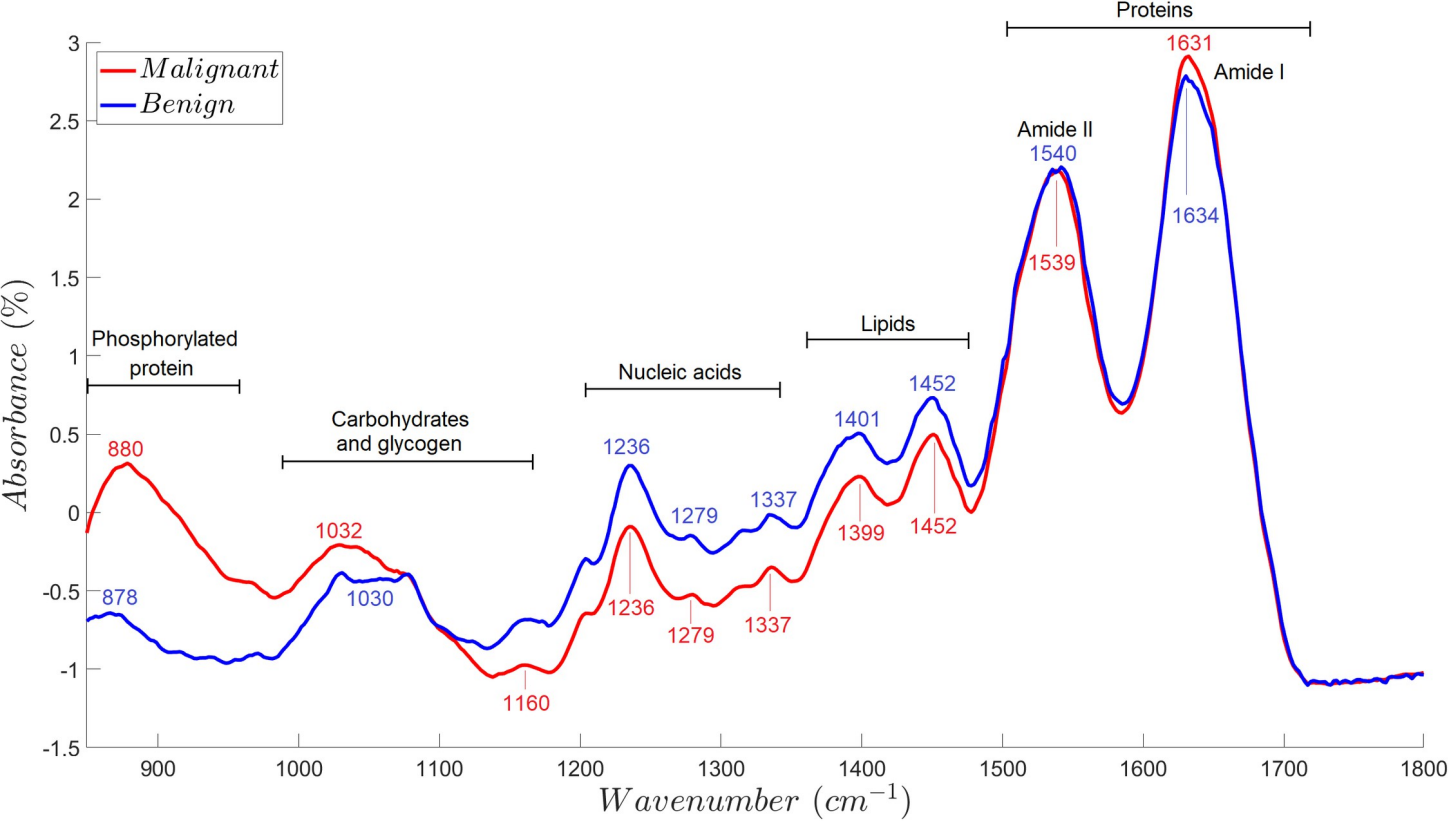
Median ATR-FTIR absorbance spectra of malignant (*n = 88*) and benign (*n = 78*) breast tissue samples. The figure shows the median FTIR spectrum of malignant and benign breast tissue samples and their corresponding peaks identified via visual analysis. The plot shows almost similar absorbance among benign and malignant samples within wavenumbers associated with the amide proteins. Benign tissue samples, relative to malignant tissue samples, are shown to have increased absorbance within the region associated with lipids and nucleic acids while having decreased absorbance within the region associated with carbohydrates, glycogen, and phosphorylated proteins.

The fingerprint spectral region (1800 cm^-1^ to 850 cm^-1^) showed that the absorbance spectra of the malignant and benign tissues were significantly distinct from each other, specifically at bands 1452cm^-1^/1452 cm^-1^, 1399 cm^-1^/1401 cm^-1^, 1337 cm^-1^/1337 cm^-1^, 1279 cm^-1^/1279 cm^-1^, 1236 cm^-1^/1236 cm^-1^, which represent lipids, DNA, RNA and phospholipids (Table 2). Other peaks tested, in particular 1632cm^-1^/1634 cm^-1^, 1539cm^-1^/1540 cm^-1^, 1160cm^-1^/1160 cm^-1^, 1032cm^-1^/1030 cm^-1^, and 880cm^-1^/878 cm^-1^, were found as non-distinct among samples. These bands represent amide I proteins, amide II proteins, carbohydrates, glycogen, and phosphorylated proteins. It is worth mentioning that the tissues analyzed had uniform thickness (5μm) achieved by sectioning with a microtome. The median absorbance spectra of benign and malignant breast tissue samples are shown in Fig 4.

Test of homogeneity showed that the characteristic IR absorbance peaks of the malignant cases were significantly different (*p*<0.05) from the benign samples. The visually identified peak positions and absorbances in the fingerprint IR region (1800 cm^-1^ to 850 cm^-1^) that could significantly differentiate the malignant from benign samples are summarized in Table 5. Their corresponding functional group, vibrational mode, and molecular source assignments are also listed in the aforementioned table. It was observed that peak absorbances representing lipids, DNA, RNA and phospholipids were significantly decreased in most malignant tissues.

**Table 5:** Comparison of the spectrum variables (peak positions and normalized absorbances) of malignant and benign breast samples in the fingerprint IR region (1800cm^-1^ to 850cm^-1^) via visual peak identification.

Malignant samples (*n* = 88)		Benign samples (*n* = 78)					*p-*value*
Peak position	Mean abs ±SD	Peak position	Mean abs ±SD	Functional Group	Vibrational Mode	Molecular Source[1–5]	
P1632	2.8311 ±0.2347	P1634	2.7934 ±0.1968	O = C-N-H	ν(CO), ν(CN)	Amide I, protein	*0*.*0553*
P1539	2.1152 ±0.2475	P1540	2.1533 ±0.1955	O = C-N-H	γ(N-H), ν(C-C), ν(C-N)	Amide II, protein	*0*.*4146*
**P1452**	**0.4797 ±0.2924**	**P1452**	**0.5920 ±0.2651**	**-(CH**_**2**_ **)**_**n**_ **, -(CH**_**3**_ **)**_**n**_ **-**	**δ**_**as**_ **(CH**_**3**_ **), δ**_**as**_ **(CH**_**2**_ **), δ**_**s**_ **(CH**_**3**_ **)**	**Lipids**	***0*.*0031***
**P1399**	**0.2208 ±0.2822**	**P1401**	**0.3375 ±0.2481**	**-(CH**_**2**_ **)**_**n**_ **-**	**δ**_**s**_ **(CH**_**3**_ **)**	**Lipids**	***0*.*0026***
**P1337**	**-0.2794 ±0.3509**	**P1337**	**-0.1598 ±0.3269**				***0*.*0140***
**P1279**	**-0.4509 ±0.3683**	**P1279**	**-0.3397 ±0.3479**				***0*.*0231***
**P1236**	**-0.0416 ±0.4474**	**P1236**	**0.1160 ±0.4204**	**RO-PO**_**2**_ **-OR**	**ν**_**as**_ **(PO**_**2**_^**-**^ **)**	**DNA, RNA, phospholipids**	***0*.*0129***
P1160	-0.8352 ±0.2566	P1160	-0.7759 ±0.2634	C-O-H	ν(CO), γ(COH)	Carbohydrates	*0*.*0673*
P1032	-0.1732 ±0.3355	P1030	-0.2386 ±0.2966	C-O-H	def(CHO)	Glycogen	*0*.*1339*
P880	0.1482 ±1.0361	P878	-0.0945 ±0.9590	C-O-P	ν(COP)	Phosphorylated protein	*0*.*0773*

* Mann-Whitney *U* test (two-tailed); significant when *p*<0.05.

**values in bold refer to significantly higher peak absorbance (*p*<0.05).

**Abbreviations**: *ν*: stretching; *δ*: bending; *γ*: wagging, twisting and rocking; *s*: symmetric; *as*: asymmetric; *def*: deformation.

**References**:

[1] Wu M, Zhang W, Tian P, et al. Intraoperative diagnosis of thyroid diseases by fourier transform infrared spectroscopy based on support vector machine. *Int J Clin Exp Med* 2016; 9: 2351–2358.

[2] Dong L, Sun X, Chao Z, et al. Evaluation of FTIR spectroscopy as diagnostic tool for colorectal cancer using spectral analysis. *Spectrochim Acta—Part A Mol Biomol Spectrosc* 2014; 122: 288–294.

[3] Simonova D, Karamancheva I. Application of Fourier transform infrared spectroscopy for tumor diagnosis. *Biotechnol Biotechnol Equip* 2013; 27: 4200–4207.

[4] Lewis PD, Lewis KE, Ghosal R, et al. Evaluation of FTIR Spectroscopy as a diagnostic tool for lung cancer using sputum. *BMC Cancer* 2010; 10: 640.

[5] Zhang X, Xu Y, Zhang Y, et al. Intraoperative detection of thyroid carcinoma by fourier transform infrared spectrometry. *J Surg Res* 2011; 171: 650–656.

The results of the performed test of significance is further backed-up in Fig 2, where wavenumbers associated with significant peak absorbances were more closely projected to *F*_1_ than those which were not. This implies that lipids, DNA, RNA and phospholipids are responsible for the high variability among the samples, suggesting further that these biomolecules highly varies between malignant and benign classes.

### Significant peaks identified by neural networks

**Fig 5:**
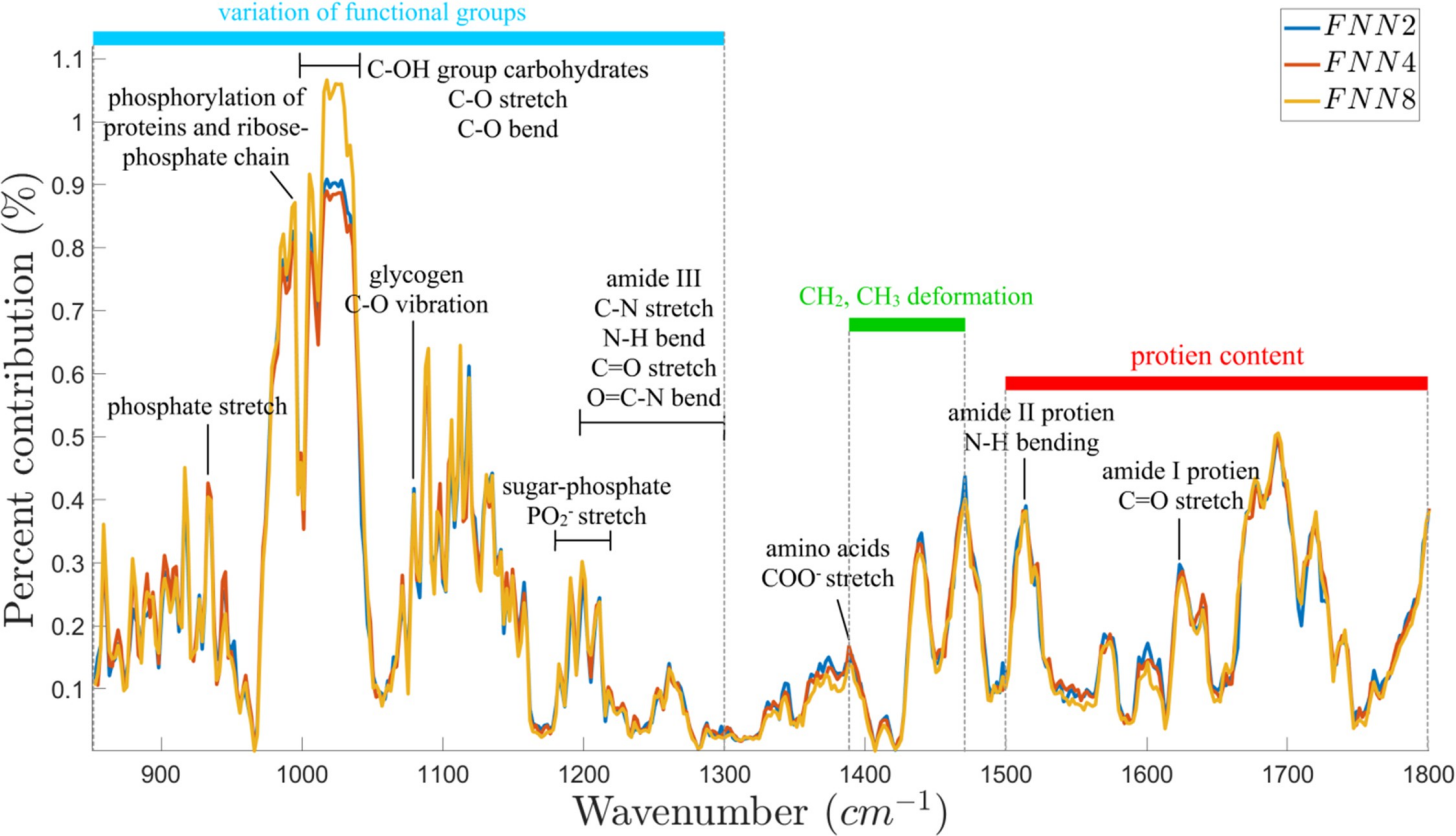
Input response of neural networks from the designed NN-based sensitivity analysis. The line plots show the input response of each neural network design per change of absorbance value per wavenumbers. A high per cent contribution magnitude implies a high response to a change for the particular wavenumber, hence may serve as a marker in identifying malignant samples from benign samples. As evident from the figure, the response of each network is nearly the same, which only varies slightly in the magnitude contribution.

The input response produced by each NN design is summarized in Fig 5. As shown, the response of each network is highly similar to one another. For all NN designs, the IR region associated with the functional groups, in particular within ~960 cm^-1^ to ~1050 cm^-1^, showed the greatest input response within the considered spectrum. Meanwhile, the least response was evident within ~1250 cm^-1^ to ~1320 cm^-1^.

Using the input response of NN from neural network committee-based sensitivity analysis, the significant peaks were identified from the fingerprint spectral region (1800 cm^-1^ to 850 cm^-1^) as shown in Fig 5. The information on the protein content, including its secondary structures such as amide I and amide II, are observed in the region between 1800 cm^-1^ to 1500 cm^-1^ [48, 49]. The bands found at ~1635 cm^-1^ are associated with the amide I protein that arises from the C = O stretching vibrations of the amide groups of the protein backbone [49, 50]. The bands at 1540 cm^-1^ results to N-H bending in the amide II groups, which is associated with aromatic amino acids. The bands 1454 cm^-1^ and 1393 cm^-1^ are associated with the CH_2,_ CH_3_ deformation modes mainly from proteins and lipids [49, 51]. Peak position 1393 cm^-1^ resulted from COO^−^symmetric stretching of amino acids [49]. The region of 1300 cm^-1^–800 cm^-1^ corresponds to the variations of functional groups that are present in proteins, nucleic acids, carbohydrates and phospholipids such as PO_2_–, C–O, and C–C [48, 52]. The band at 1238 cm^-1^ is at the range of sugar-phosphate chain vibrations which is related to PO_2_^−^ asymmetric stretching of nucleic acids [52]. Furthermore, this is also the range for Amide III (1299 cm^-1^–1200 cm^-1^) for its C-N stretching, N-H bending, C = O stretching, and O = C-N bending [53, 54]. The vibrations of C–O group from glycogen are observed in peak 1077 cm^-1^ [51]. The band 1030 cm^-1^–1045 cm^-1^ is due to C–O stretching and C–O bending of the C–OH groups of carbohydrates such as glucose, fructose [50, 54]. The band at 990 cm^-1^ is related to phosphorylation of proteins and ribose-phosphate chain [50] while ~962 cm^-1^ is associated with symmetric phosphate stretching modes from phosphate diester groups in nucleic acids and phospholipids [51].

## Discussion

FTIR spectroscopy is a prospective novel diagnostic tool that is used to distinguish cancer samples from normal ones at high sensitivity, specificity, and accuracy [55, 56]. Considering the molecular complexity of biological specimens, chemometric techniques such as the principal component analysis (PCA) and artificial neural networks (ANN) that combine statistical and mathematical algorithms are utilized to generate chemo-physical evidence from spectral data [55].

The advent of computers with enhanced processing capabilities and enhanced memory capacity have led in the rise of computer-aided diagnosis (CAD), which combines algorithms or methods from pattern recognition and digital image processing [56]. Meanwhile, scientists have been drawn to the potential application of FTIR spectroscopy in the clinical setting to improve accuracy and reproducibility of cancer diagnosis, while omitting the need for complex and time-consuming clinical processing of tissue biopsy samples [55]. This is best exemplified by the study of Großerueschkamp, *et al*., wherein they combined FTIR imaging and a novel trained random forest (RF) classifier for the automated marker-free histopathological annotation of lung tumor classes and subtypes of adenocarcinoma without further treatment of tissue samples. This study yielded greater reproducibility and accuracy of 97% for the annotation of lung tumor classes and 95% for the identification of prognostic adenocarcinoma subtypes [57]. Subsequently, FTIR reduced intra- and inter-operator variability through its objectivity, reproducibility, and improved accuracy over current methodologies for cancer diagnosis [55]. This also permits the standardization of spectral measurement and analysis, which is necessary for the construction of FTIR spectral databases with highly specific spectroscopic markers for the various stages and grades of different cancer types applicable to the clinical settings [58]. Additionally, an easy and objective data interpretation can be done by non-spectroscopists by incorporating powerful algorithms for automatic data analysis of large data sets [55].

The designed NNs exhibited superior accuracy (˃90%) relative to the best benchmark model (SVM). These metrics prove them not only as excellent classifiers in distinguishing malignant from benign breast cancers using ATR-FTIR data, but also excellent classifiers in general. Overall, the FNN2 model was able to obtain larger metric values relative to the other NN models. The decrease in the performance metrics, in particular, accuracy, NPV, and RR of the models as a function of the layer quantity makes it evident that the deeper models may have lacked training time. Do note, however, that an opposite trend is evident for the SR and PPV metrics, implying that the designed architectures may approach a classifier that becomes increasingly more accurate in detecting malignant rather than benign samples as the model gets deeper. This observation presents a trade-off between the model’s capability in confirming truly malignant from benign samples. This, further, implies that if a more accurate positive screening test is more in-demand, then deeper models may be assumed. Conversely, for more accurate negative screening tests, a less deep model may be more necessary.

Considering the metric comparison performed, the non-significant PPV metric of the designed NNs make them equally competent to SVM. However, the significantly higher NPV metric of the designed NN models make them more superior classifiers in terms of identifying benign samples as truly exhibiting non-malignancy. The significance of the SR and the RR are parallel to the significance of the PPV and NPV by definition, respectively. This characteristic makes the designed NN models more practical to use in situations where diagnosing non-malignant patients as malignant becomes very costly. While administering an incorrect diagnosis is very detrimental for a patient in general, for financially non-capable individuals, an accurate diagnosis of non-malignancy may be of more importance since a false diagnosis of malignancy risks the individual of financial burden in chemotherapy, and a probable decline in health which further necessitates added costs. In developing countries such as the Philippines, the use of highly specific diagnostic tool such as the designed NNs in this study may prove more beneficial for patients undergoing cancer diagnosis. Regardless of the use, however, the models show their potential as highly specific tools to assist pathologists and medical practitioners in the field.

The designed neural networks that were used to analyze the FTIR spectra were able to identify significantly decreased peak absorbances characteristic of lipids, nucleic acids, and phospholipids in malignant tissues, which were similarly evident in the performed visual analysis (Table 5). Breast cancer is often characterized by the stimulated production of novo lipids which are essential for cell growth, proliferation, and oxidative stress resistance. The triacylglyceride storage in lipid droplets has been suggested to work as fuel source after re-oxygenation during intermittent hypoxia, whereas fatty acids promote redox balance supporting a high-glycolytic rate in malignant tissues. Lipids also form the structural basis of paracrine hormones and growth factors which stimulate tumor growth, neovascularization, invasion, and metastatic spread [59]. The decrease in lipids and phospholipids may reflect the utilization of these biomolecules for nutrition and energy source; and thus, prevent there accumulation in cancer cells during cancer progression [60]. A significant difference in the absorptive peak was also apparent in the DNA/RNA spectral region, with the malignant breast samples showing significantly lower peak absorbance than benign samples. This is in contrary to the findings of Lazaro-Pacheco *et al*. wherein higher contribution of nucleic acid bands was identified in cancerous samples in comparison with normal breast tissues in different spectral regions. They argued that high concentration of these biomolecules is expected since there is increased cellular content in response to an abnormal proliferation [61]. However, in a study involving ovarian cancer, the RNA/DNA absorption peaks were significantly lower in malignant tissues than in borderline and benign ovarian tissues [62]. The lowered peak absorbance among malignant samples may be due to fragment transfer of tumor DNA or cell-free RNA from the cancerous area to the bloodstream; thus, consequently decreasing the nucleic acid content at the primary tumor area [63].

Interestingly, there was no distinctive difference between malignant and benign breast tissues in the absorptive peaks of carbohydrates as perceived by the visual analysis performed (Table 5). In relation, the P1160 wavenumber vector that is associated with carbohydrates was projected significantly far from *F*_1_, implying further that this biomolecule is not a possible cause of variability (Fig 2). However, the sensitivity analysis stated otherwise since the highest peak was evident across the IR region associated with the C–OH group of carbohydrates (Fig 5). Studies have shown that assessing glycogen levels is a good differentiation marker between malignant and benign tissues, with malignant samples generally consuming more glycogen to sustain survival during prolonged hypoxia and glucose deprivation as well as to sustain metastasis [64]. This ability of the sensitivity analysis to recognize the carbohydrates as differentiating factor, which in contrast was not detected by mere visual peak analysis, further proves the proposed method as a more discerning method to identify important spectral biomarkers. Possibly, the breast cancer cells could have already catabolized their glycogen stores as well as their subsequent by-products such as glucose for survival in nutrient-deprived environment [23]; hence, became relatively indistinguishable by the usual visual peak analysis. Given the proposed method’s superior ability, the study suggests that the identified peaks within the higher IR wavelength region (~1400 cm^-1^ to ~1800 cm^-1^) be given attention, particularly those associated with CH_2,_ CH_3_ deformation modes and amide protein stretch and bends.

A variety of biological materials including blood, solid tissues, urine, and sputum have been studied using FTIR spectroscopy to develop better alternatives for cancer diagnosis and management. In the clinical setting, blood and tissue samples remain to be widely used as opposed to other specimens for diagnosing disease [55]. In less developed countries, the use of FFPE samples can provide technical ease and economic advantage for longitudinal tissue specimen storage as they can be easily retrievable from accredited repositories for further analysis [65]. Compared to immunohistochemical and molecular assays, FTIR can be a cheaper alternative for detecting biochemical markers in pathologic FFPE specimens based on unique vibrational patterns [60]. With the introduction of machine learning, FTIR spectroscopy in clinical diagnostic settings can reduce intra- and inter-operator variability and improve accuracy and reproducibility of cancer diagnosis, while omitting the need for complex and time-consuming clinical processing of clinical samples [56].

The generation of NN models from the FTIR fingerprint of benign and malignant FFPE breast tissues led to the identification of significant wavenumbers apart from those at peak absorbances, which can be used to discriminate malignant from benign tissues. Interestingly, unique peak absorbances distinctive of lipids, nucleic acids, and phospholipids were identified, showing that these biomolecules were significantly decreased in malignant tissues as compared to benign samples, and can, therefore, be used as biochemical fingerprints to aid in cancer diagnosis.

While the current study shows that NN models from FTIR spectra can be used as an adjunct tool for diagnosing breast cancer, additional clinical studies should be made to bring this technology into the clinical setting. Due to financial constraint, this study was conducted using only the basic type of FTIR spectrometer with limited spatial resolution. To acquire a comprehensive spectral data, additional FFPE samples may be analyzed using an FTIR coupled with an infrared microscope to detect vibrational motions of molecules within very restricted regions. Other spectroscopic techniques such as the Raman spectroscopy can also be used to further probe molecular vibrations to aid in the characterization and discrimination of tissue types [66]. The creation of spectral database and the generation of novel powerful algorithms for automatic data analysis of large data sets is another prospect to accelerate point-of-care decisions and improve therapeutic management for breast cancer patients [67]. Further studies also show that an alternative sample to tissues could be blood plasma, since the use of plasma is cheaper, less invasive, and easier to process [68, 69]. Through the integration of AI and FTIR, spectral biomarkers in plasma samples may be identified to monitor treatment response; a study which is already being investigated by the research team.

In summary, the present study generated NN models that led to the identification of unique infrared spectrum of absorption in the lipid, nucleic acid, phospholipid, and carbohydrates regions that could effectively discriminate malignant from benign breast tissues. To the researchers’ knowledge, this is the first study to have used several machine learning tools to identify malignant breast tissues based on FTIR spectral data.
